# Extensive sampling of polar bears (*Ursus maritimus*) in the Northwest Passage (Canadian Arctic Archipelago) reveals population differentiation across multiple spatial and temporal scales

**DOI:** 10.1002/ece3.662

**Published:** 2013-08-03

**Authors:** Leonardo Campagna, Peter J Van Coeverden de Groot, Brenda L Saunders, Stephen N Atkinson, Diana S Weber, Markus G Dyck, Peter T Boag, Stephen C Lougheed

**Affiliations:** 1 Department of Biology, Queen's UniversityKingston, Ontario, Canada, K7L 3N6; 2 División de Ornitología, Museo Argentino de Ciencias Naturales “Bernardino Rivadavia”Avenida. Ángel Gallardo 470, Ciudad de Buenos Aires, Buenos Aires, Argentina, C1405DJR; 3 53 Ashland Ave., Winnipeg, Manitoba, Canada, R3L 1K3; 4 Division of Natural Sciences, New College of Florida5800 Bay Shore Road, Sarasota, Florida; 5 Department of Environment, Government of NunavutIgloolik, Nunavut, Canada, X0A 0L0

**Keywords:** Conservation genetics, DNA microsatellites, marine mammals, mark-recapture, mitochondrial DNA, species at risk

## Abstract

As global warming accelerates the melting of Arctic sea ice, polar bears (*Ursus maritimus*) must adapt to a rapidly changing landscape. This process will necessarily alter the species distribution together with population dynamics and structure. Detailed knowledge of these changes is crucial to delineating conservation priorities. Here, we sampled 361 polar bears from across the center of the Canadian Arctic Archipelago spanning the Gulf of Boothia (GB) and M'Clintock Channel (MC). We use DNA microsatellites and mitochondrial control region sequences to quantify genetic differentiation, estimate gene flow, and infer population history. Two populations, roughly coincident with GB and MC, are significantly differentiated at both nuclear (*F*_ST_ = 0.01) and mitochondrial (Φ_ST_ = 0.47; *F*_ST_ = 0.29) loci, allowing Bayesian clustering analyses to assign individuals to either group. Our data imply that the causes of the mitochondrial and nuclear genetic patterns differ. Analysis of mtDNA reveals the matrilineal structure dates at least to the Holocene, and is common to individuals throughout the species’ range. These mtDNA differences probably reflect both genetic drift and historical colonization dynamics. In contrast, the differentiation inferred from microsatellites is only on the scale of hundreds of years, possibly reflecting contemporary impediments to gene flow. Taken together, our data suggest that gene flow is insufficient to homogenize the GB and MC populations and support the designation of GB and MC as separate polar bear conservation units. Our study also provide a striking example of how nuclear DNA and mtDNA capture different aspects of a species demographic history.

## Introduction

Climate change is expected to have a profound impact on polar bears (*Ursus maritimus*), affecting the spatial and seasonal distribution of the sea ice that they rely on to travel and hunt (Stirling and Derocher 1993; Derocher et al. 2004). During the 21st century the availability of suitable polar bear habitat is predicted to decline, with a potential negative effect on the species abundance (Amstrup et al. 2008; Durner et al. 2009). Adaptation to this rapidly changing environment, should it occur, will necessarily involve changes to the spatial distribution of individuals. This may result in range shifting or local extinction of some populations. The design of cogent and effective conservation strategies requires a detailed understanding of how the population dynamics are changing. This is of even greater importance if traditional harvesting practices of the species are to continue on a presumed sustainable basis (Lee and Taylor 1994; Schliebe et al. 2008).

Currently, 19 distinct polar bear populations are recognized worldwide (Obbard et al. 2010; see Paetkau et al. 1999 for a detailed map of the geographic distributions of these populations), established on the basis of land barriers and movement patterns inferred through mark-recapture and radiotelemetry data (Taylor and Lee 1995; Bethke et al. 1996; Taylor et al. 2001). These 19 populations constitute the management units that are used to delineate global conservation strategies and establish harvest quotas. Evidence from genetic data also supports the designation of the majority of these populations (Paetkau et al. 1995, 1999). In the most geographically comprehensive study on polar bear genetics performed to date, Paetkau et al. (1999) used 16 microsatellite loci to survey 16 of the 19 recognized polar bear populations. Although there were significant genetic differences for most pairwise population comparisons, these were generally small with no striking discontinuities across the range. Populations clustered into four groups suggesting some overall genetic structure. Paetkau et al. (1999) interpreted these differences as the product of contemporary movement patterns, possibly reflecting the configuration of land masses in conjunction with the seasonal distribution of sea ice and how this enables access to seals.

**Figure 1:**
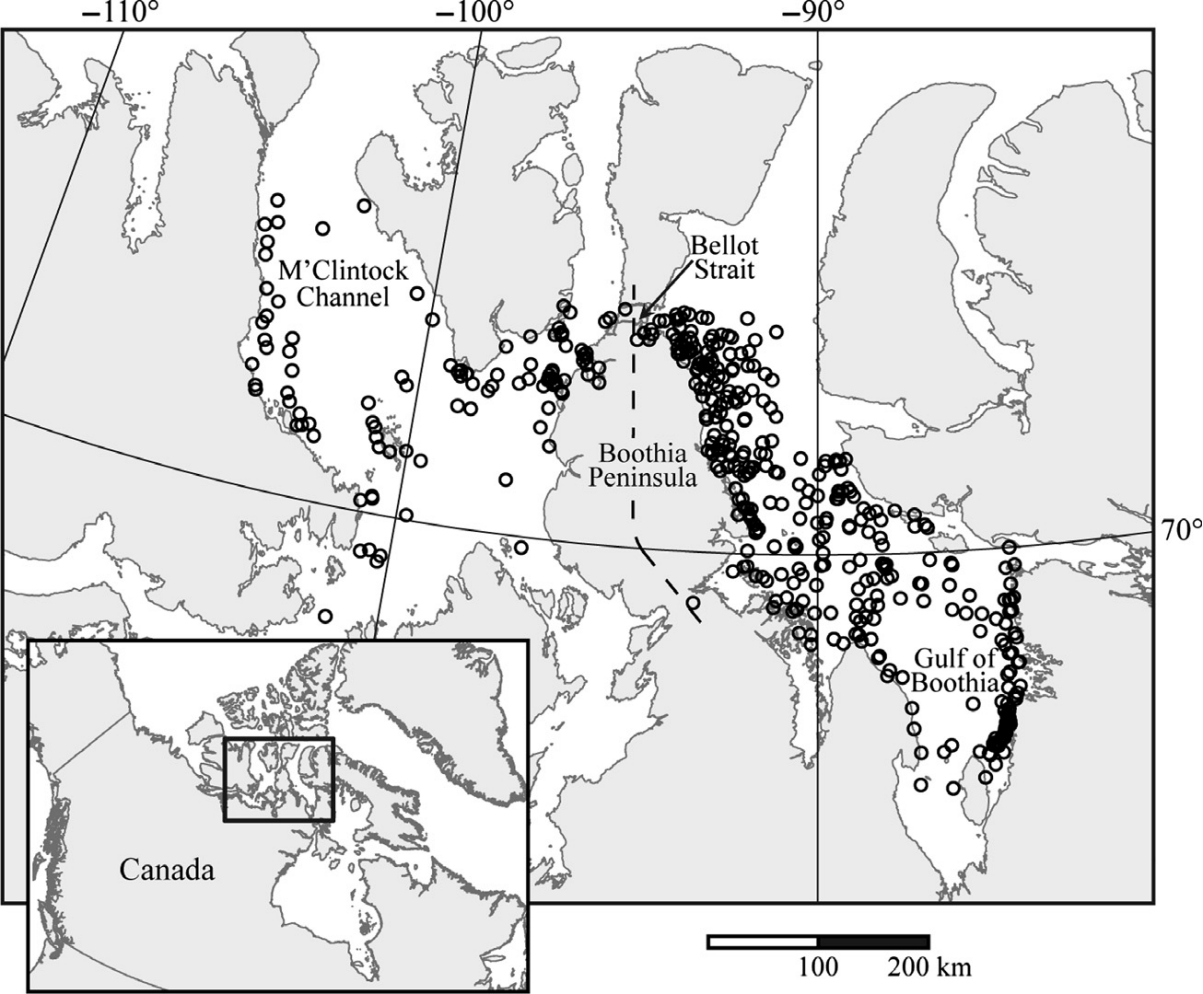
Map of the study area showing the locations where each of the 718 polar bears where captured. The dotted line represents the border between the Gulf of Boothia and M’Clintock Channel populations, designated through previous studies of polar bear movement patterns.

The study by Paetkau et al. (1999) focused on obtaining samples from nearly all polar bear populations, thus providing insight into the genetic structure across the species’ range. An alternative strategy, which targets a small number of populations with continuous and intensive sampling (e.g., Cronin et al. 2006; Crompton et al. 2008; Zeyl et al. 2009), allows us to characterize genetic structure in greater detail and assess how these groups are or have been connected and perhaps how they arose. Here, we focus on two previously designated polar bear populations geographically located in the center of the Canadian Arctic Archipelago: the Gulf of Boothia (GB), which encompasses an area of ∼170,000 km^2^; and the adjacent M'Clintock Channel (MC), that extends over ∼500,000 km^2^. These areas are separated by a land barrier (the Boothia Peninsula) for the majority of their length and connected by water only through the narrow Bellot Strait, an ∼25 km long passage with fast tidal currents that prevent the formation of stable pack ice (Fig. 1). While the GB polar bear population has been suggested to be demographically stable, the MC population is increasing in size due to management measures that have allowed recovery from intense hunting pressure (Obbard et al. 2010). These populations are situated within the Northwest Passage, a new navigation route that will increasingly facilitate shipping across the Arctic seas along the top of North America as sea ice continues to melt (Kerr 2002). This expanding development of the Arctic puts the GB and MC polar bear populations at risk of being significantly disturbed by anthropogenic stressors (e.g., increased pollution or direct interactions with humans) in the near future (Amstrup et al. 2008; Obbard et al. 2010). In our study, we use mitochondrial and nuclear DNA sequences combined with extensive sampling to address the following questions:

1. To what extent are the GB and MC populations genetically differentiated? If significant genetic differences do exist, over what time frame did they arise and what might have been the cause?
2. Does gene flow occur between regions and if so, is there evidence of sex-biased gene flow? Do diagnosed patterns of gene flow match our knowledge of movement patterns deduced from mark-recapture data?

## Material and Methods

### Sampling and data set

Samples for genetic analyses were collected by the Nunavut Department of Environment during surveys carried out in the spring (March–June) between 1998 and 2000 in two previously defined polar bear populations: GB and MC (Fig. 1). Part of this region is characterized by perennial ice where polar bears remain throughout the year (Amstrup et al. 2008; see Barber and Iacozza (2004) for a detailed analysis of the annual sea ice patterns in the study area). After bears were immobilized, capture site and sex were recorded, age was estimated and a tissue sample (a small disk of skin obtained from ear tagging) was taken for DNA analysis. For details on sampling procedures, see Taylor et al. (2006, 2009). In total our data set contains 718 samples, including mothers with their cub(s) and solitary males; some individuals were captured up to three times (during each of the 3 years of fieldwork). For genetic analyses we used 361 unique male and female adult and subadult individuals (GB, *N* = 289; MC, *N* = 72), discarding genetic data from cubs, yearlings and second-year bears that were related to the sampled mother.

We used mark-recapture data to compare movement patterns between sexes and populations. Movement was estimated by calculating the straight-line distance between sites where each individual was captured in different years using the great circle distance calculator in Google Maps. When a bear was captured in three different years, we only used the maximum distance between capture sites to avoid pseudo-replication. Three females moved from MC to GB and could not be a priori assigned to either population (and thus be confidently used to compare movement patterns between GB and MC). We analyzed the data in two ways, first excluding these three females and then again considering them part of the population where they were initially captured (MC). In total we obtained 56 independent observations of distances traveled by individual bears during the study period. We compared movement patterns between sexes and populations by performing a two factor analysis of variance (ANOVA) in *JMP* version 10 (SAS Institute Inc., Cary, NC).

### Microsatellite amplification and genotyping

Genomic DNA was extracted from a small disk of skin using the QIAGEN DNA extraction kit (QIAGEN, Mississauga, Canada) following the manufacturer's procedures. We genotyped 531 individuals (including 361 adults/subadults, and an additional 170 cubs, yearlings and second-year bears for maternity analysis) for nine previously published dinucleotide microsatellite loci (G1A, G1D, G10B, and G10L: Paetkau and Strobeck 1994; G10M, G10P, and G10X: Paetkau et al. 1995; MU59: Taberlet et al. 1997; G10H: Paetkau et al. 1998), following protocols outlined in Saunders (2005). Random subsets of individuals were genotyped more than once to confirm repeatability of results. We used the family groups in our data set to evaluate possible genotyping errors by performing a maternity analysis with *Cervus* version 3.0.3 (Kalinowski et al. 2007). This data set includes 170 cubs belonging to 108 known mothers, thus totaling 2502 genotypes across our nine loci. The maternity analysis revealed 24 single locus genotypes (0.96% averaged across all genotypes or 2.14% averaged across loci) that did not match between cubs and their mothers. One mismatch could have been caused by null alleles, but the remaining 23 could be due to either mutation or genotyping error. Regardless, we were confident that this low rate would have negligible impact on our results. Adult and subadult individuals were used to test for Hardy–Weinberg equilibrium and linkage disequilibrium in *Arlequin* version 3.5.1.3 (Excoffier and Lischer 2010); we excluded all cubs to reduce biases due to inclusion of related individuals. The nine loci show no deviations from Hardy–Weinberg expectations, both when individuals from GB and MC were analyzed together and separately. When GB and MC individuals were analyzed together, the locus pairs G10M/MU59 and G10B/G10H showed significant linkage desequilibrium after sequential Bonferroni correction (Rice 1989). When GB and MC samples were analyzed separately, only G10B and G10H showed significant linkage disequilibrium in the GB population, perhaps a consequence of admixture between genetically distinct individuals occurring within GB.

### Mitochondrial control region amplification and sequencing

We selected a subset of 86 subadult and adult individuals evenly distributed throughout the study area (GB, *N* = 48; MC, *N* = 38) for the amplification and sequencing of an ∼472 base pair (bp) fragment of the mitochondrial control region (CR; positions 16,545–17,018 in the polar bear mitochondrial genome reference sequence AF303111 deposited in GenBank). Amplification was carried out using primers PBCR1 (5’-AGCTCCACTACCAGCACCC-3’) and PBCR 4 (5’-AAATGCATGACACCACAGTTATGTGTGATC-3’) (Weber et al. unpubl. ms.). Polymerase chain reaction (PCR) cocktails contained 2 μL of genomic DNA, 2.5 mmol/L MgCl_2_, 0.1 mmol/L dNTPs, 0.1 μmol/L of each primer, and 0.75 U taq DNA polymerase (Vivantis Technologies, Malaysia) in a final volume of 25 μL PCR ViBuffer A (Vivantis; 50 mmol/L KCl, 10 mmol/L Tris-HCl pH 9.1 and 0.01% Triton X-100). The thermocycling profile included an initial denaturation step at 94°C for 5 min; followed by 34 cycles of 94°C for 30 sec, 50°C for 30 sec, and 72°C for 45 sec; and a final extension at 72°C for 5 min. An aliquot of the PCR products was visualized on an ethidium bromide-stained 2% agarose gel and successful reactions were purified using the QIAquick PCR purification Kit (QIAGEN). Sequencing was carried out in both directions using the above mentioned primers at the London Regional Genomics Centre (London, Ontario, Canada) using an Applied Biosystems 3730 Analyzer. All sequences were deposited in GenBank (Accession Numbers KF192517 – KF192602).

### Measures of genetic distance

Genetic differentiation between populations was estimated with *Arlequin* version 3.5.1.3, using DNA microsatellite and CR data to calculate different F-statistics or their analogs. For DNA microsatellites, we calculated *F*_ST_ values; for the CR data we estimated differentiation using haplotype frequencies alone (*F*_ST_), or taking into account the difference among haplotypes together with their frequencies (Φ_ST_). Statistical significance was assessed through 1000 random permutations. We calculated the genetic diversity within populations by estimating p-distances in *Arlequin* and *F*_IS_ values in *FSTAT* version 2.9.3.2 (Goudet 1995), for sequence and microsatellite data, respectively. We used the program *Populations* version 1.2.32 (Langella 1999) and microsatellite data to calculate *D*_SW_ pairwise genetic distances (Shriver et al. 1995; a measure of genetic distance developed for microsatellite loci) between all individuals. The program *GenAlEx* version 6 (Peakall and Smouse 2006) was used to calculate geographic distances between individuals from geographic coordinates of capture sites. We tested for the effect of individual-based isolation by distance (Rousset 2000) by performing a Mantel test (Mantel 1967) in *GenAlEx*, with the null hypothesis of independence between the genetic and geographic distance matrices, and assessing significance through 999 random permutations. We analyzed the data separately for males, females, and both sexes pooled, and performed these analyses for GB and MC separately, and for both regions pooled. Because nine comparisons were carried out sequential Bonferroni corrections were applied to minimize type I errors.

### Bayesian clustering analyses

We assessed population structure using individual genotypes for the nine microsatellite loci and two programs that implement Bayesian clustering algorithms: *Structure* version 2.3.4 (Pritchard et al. 2000) and *Geneland* version 4.0.3 (Guillot et al. 2005). Analyses were conducted for males and females separately and pooled. *Structure* was run using the admixture ancestry model, correlated allele frequencies and both with and without *LOCPRIOR* (i.e., a prior that indicates the population sampling origin). We explored values of *K* = 1 through 4 (two more than the a priori reported number of populations) with ten iterations per value of *K* each with 2,500,000 generations, discarding the initial 500,000 as burn-in. The most likely *K* value was determined using the Evanno et al. (2005) method implemented in *Structure Harvester* version 0.6.93 (Earl and vonHoldt 2012), and by inspecting individual assignment patterns (specifically when comparing between results from *K* = 1 and *K* = 2, for which the Evanno et al. method cannot be applied).

**Figure 2:**
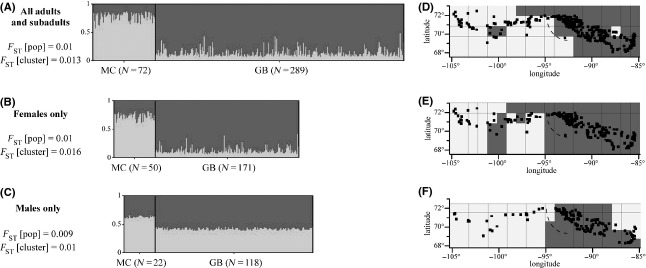
Results from Bayesian clustering analyses performed in *Structure* and *Geneland* using data from nine DNA microsatellite loci. *Structure* bar plots representing assignment of adult and subadult individuals (A), females (B), or males (C) from GB and MC to one of two genetic populations (represented by light and dark gray bars). Admixed individuals are represented by bars with varying proportions of light and dark gray (scale on the *y-*axis). Maps of the study area divided in quadrats of ∼1° longitude by 1° latitude that were assigned to one of two genetic populations (light or dark gray), according to the genotypes of sampled individuals (D: adult and subadults; E: females; F: males). Black squares represent sampled individuals; thus assignment of quadrats where individuals have not been sampled represent extrapolations and must be interpreted with caution. The dotted line shows the border between the GB and MC populations. *F*_ST_ values represent comparisons between individuals from GB and MC (denoted by *F*_ST_ [pop]) calculated in *Arlequin* or degree of divergence between genetic clusters (denoted by *F*_ST_ [cluster]) calculated in *Geneland*.

We conducted two independent *Geneland* runs per group analyzed (males, females, and sexes pooled), each consisting of 2,500,000 iterations and a thinning of 100. The number of genetic populations was set to *K* = 2 based on results of *Structure* runs, using correlated allele frequencies and the spatial model. After discarding 5000 steps (25%) as burn-in, we divided the study area into 100 quadrats (20 by 5, ∼1° longitude by 1° latitude; Fig. 2D–E), to obtain a map of population membership and *F*_ST_ values between the two genetic clusters. Similar results were reached for replicate runs.

### Network and phylogenetic analyses

We aligned mitochondrial sequences using *BioEdit* version 7.0.9.0 (Hall 1999) and explored the relationship between CR haplotypes using a statistical parsimony network analysis in *TCS* version 1.21 (Clement et al. 2000). To compare the variation found in CR sequences obtained from individuals belonging to GB and MC to the overall phylogeographic structure observed in the species, we downloaded published CR sequences (Edwards et al. 2011) from Genbank. In total, we incorporated 45 sequences obtained from modern individuals sampled from across the Arctic (from 1883 to present, accession numbers: GU573485; GU573490; and sequences with locality information with numbers between JF900105-153). Sequences were aligned to our 86 CR sequences from GB and MC, creating a combined data set of 131 sequences from individuals across the species’ range with which we constructed a new statistical parsimony network. We also used this combined CR data set to build a Bayesian phylogenetic tree using *MrBayes* version 3.2.1 (Huelsenbeck and Ronquist 2001; Ronquist and Huelsenbeck 2003). The tree was rooted using a brown bear CR sequence (*Ursus arctos*: GenBank accession number EF033706). The HKY (Hasegawa et al. 1985) +G model of nucleotide evolution best fit the data according to an analysis performed in *jModeltest* version 0.1.1 (Posada 2008). Two simultaneous Bayesian searches using four incrementally heated Markov chains and default priors for all parameters were run for six million generations. At this point the standard deviation of split frequencies was <0.01, suggesting the analysis had converged. Trees were sampled every 100 generations, and we discarded the first 25% as burn-in. All parameters had a potential scale reduction factor (Gelman and Rubin 1992) that was close to one, indicating that we had adequately sampled the posterior distribution. We also assessed convergence using the “cumulative” and “compare” functions implemented in the software AWTY (Wilgenbusch et al. 2004), confirming that runs had reached stationarity. Finally, we obtained a 50% majority rule consensus from the retained, combined posterior tree distribution of both runs.

### Bayesian coalescent simulations

We used the program *IMa2* (Hey 2010), that implements the isolation with migration (IM) model, to estimate gene flow, effective population sizes, and divergence time between GB and MC. We ran the program using either mitochondrial DNA (mtDNA, CR sequences) or nuclear DNA (nuDNA, nine DNA microsatellite loci) separately, applying the HKY model to the former and the stepwise mutation model to the latter (Ohta and Kimura 1973). In both cases the IM model was simplified to include only one (bidirectional) migration rate parameter, thus reducing the total number of parameters estimated by the model. Positions in the CR sequence alignment with ambiguous bases or missing data were ignored by *IMa2*; thus the analysis was conducted using 271 bp of the 472 bp amplified originally. Runs in M mode showed adequate mixing with 100–120 chains and a burn-in of 250,000–500,000 generations for the CR and microsatellite data, respectively. We implemented the geometric heating model and ran the program at least four times, with different random seeds, until a minimum of 100,000 genealogies were saved. Joint-posterior densities of model parameters were finally estimated in L mode. Estimations of population migration rates (2*Nm*), the effective number of migrants per generation, were obtained from the migration (m/μ) and θ (4*Nμ*) parameters calculated with *IMa2*. By calculating 2*Nm* = 4*Nμ* × 1/2 × m/μ, we estimated 2*Nm* independent of the mutation rate (Hey and Nielsen 2004). Because the model was simplified to include only one bidirectional migration parameter, we used the average θ between GB and MC to calculate 2*Nm*. Finally, effective population sizes for GB and MC were also estimated using DNA microsatellite data and an approximate Bayesian computation framework in *ONeSAMP* (Tallmon et al. 2008), exploring parameter values up to each population's census size.

## Results

### Movement patterns inferred through mark-recapture data

Based on our mark-recapture data, the average distance traveled was slightly larger for males than for females (71 ± 60 km vs. 57 ± 61 km), and for MC than for GB individuals (89 ± 90 km vs. 54 ± 45 km), although these differences were not statistically significant. The maximum distance traveled was larger for females (317 km) than for males (194 km), and for MC (317 km) than from GB (185 km). In three instances, females that were initially observed within the MC boundaries were subsequently captured in the area encompassed by GB (having traveled 86, 137, or 230 km). When these three females were included in the population from which they had initially been captured (MC), MC individuals were found to have traveled larger distances compared to those of GB (101 ± 88 km vs. 54 ± 45 km), and this difference was statistically significant (two factor ANOVA: *F*_1, 52_ = 6.310, *P* = 0.015).

### Nuclear differentiation between individuals from GB and MC

Bayesian clustering analyses performed using data from nine microsatellite loci suggest GB and MC polar bear populations are genetically differentiated (Fig. 2). The most likely number of genetic populations (*K*) identified by *Structure* was two, corresponding to differentiation between the GB and MC populations, although individuals show varying levels of admixture (Fig. 2A). Using the Evanno et al. (2005) method, we did not find support for values of *K* greater than 2, and likelihood scores were slightly larger for *K* = 1 than *K* = 2 (see Appendix S1). However, for *K* = 2 *Structure* assigned ∼90% of the membership of GB and ∼80% of MC into two distinct clusters (Fig. 2A and Appendix S1), suggesting genetic structure can be detected between these populations. The differentiation was weak, although statistically significant (*F*_ST_ = 0.01, *P* < 0.0001), causing *Structure* to fail in assigning individuals to either genetic population without incorporating sampling locality as a prior in the Bayesian analysis. Using information from individual genotypes and sampling coordinates, *Geneland* assigned most quadrats in the GB to the GB genetic cluster, and most MC quadrats to the MC cluster, with an *F*_ST_ value of 0.013 between clusters (Fig. 2D). However, as a consequence of the apparent admixed ancestry of some individuals, a few quadrats within the GB were more closely allied to the MC cluster, and vice versa (Fig. 2D). We found no isolation by distance, either when all samples were analyzed together or when GB and MC samples were analyzed separately. Note that not every quadrat was sampled (Fig. 2D–F), and thus the assignment of such “empty” quadrats represents an extrapolation that must be interpreted with caution. Moreover, the genetic identity of quadrats within populations must be interpreted as transitory. *Geneland* performs these assignments using the coordinates where the individual was sampled and it is likely that polar bears move freely within GB and MC. Moreover, this analysis was conducted pooling individuals from three different years and our mark-recapture data suggest that average movements on the order of 50–70 km between years are common. Thus our *Geneland* results are approximate and it is likely that the genetic identity of quadrats within GB and MC changes as bears move on the landscape. Finally, the *F*_IS_ value was slightly larger in MC than in GB (0.012 vs. −0.001). Altogether, these results suggest nuclear genetic differentiation between GB and MC with a low level of admixture.

We tested for sex-specific patterns in DNA microsatellites by performing the same analyses as above for each sex separately. Again *Structure* assigned individuals to two genetic clusters corresponding to GB and MC (when capture location was used as a prior); however, differentiation was higher in females than males (Fig. 2B vs. 2C). *F*_ST_ values between GB and MC individuals and between diagnosed genetic clusters (i.e., the differentiation between light and dark gray quadrats identified by *Geneland*) were both marginally higher for females than for males (0.01 vs. 0.009 and 0.016 vs. 0.01, respectively). As before, for both sexes *Geneland* assigned most quadrats in GB to a different genetic cluster than those in MC and vice versa (Fig. 2E and F). Again, we found no significant isolation by distance for males or females overall or analyzing GB and MC individuals separately. Although sample sizes are lower for males compromising statistical power, these results are consistent with males exhibiting higher dispersal between populations.

### Mitochondrial differentiation between individuals from GB and MC

**Figure 3:**
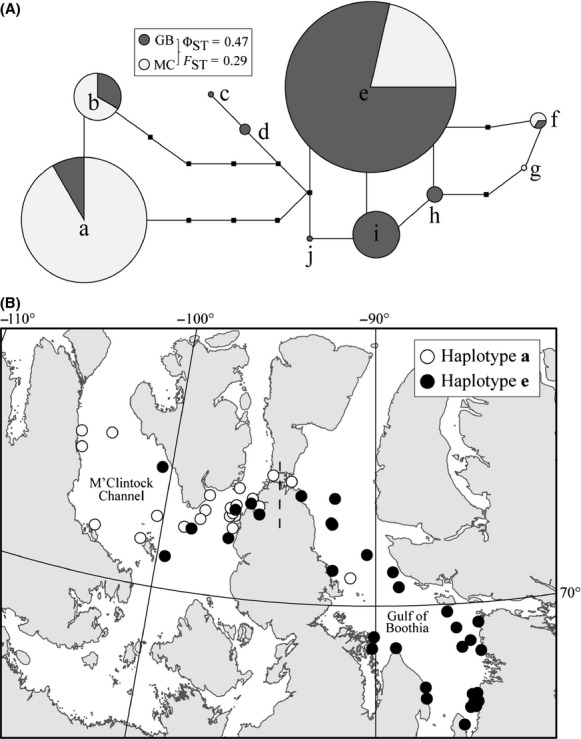
Statistical parsimony network analysis performed with CR sequences from 86 polar bear individuals. (A) Maximum parsimony network showing 95% probability linkages among 10 CR haplotypes. Lines represent mutational changes with black squares indicating hypothetical haplotypes. The area of each circle is proportional to the number of individuals with that haplotype: the smallest circles are haplotypes found in one individual while the largest represents 33 individuals. Haplotypes are coded in dark or light gray following the populations where they were sampled (GB or MC, respectively). (B) Map representing the sampling location of individuals carrying the two most common haplotypes (*a* and *e*) which differ in frequency across populations.

The CR statistical parsimony network analysis implies genealogical distinction between GB and MC (Fig. 3). The network analysis revealed 10 CR haplotypes (labeled *a*–*j*) among 86 individuals that differed from each other by up to nine mutational steps (Fig. 3A). Haplotypes and their frequencies differed significantly between GB and MC (Φ_ST_ = 0.47 and *F*_ST_ = 0.29, *P* < 0.001), with haplotype *e* being most common among GB individuals and haplotype *a* most abundant among MC individuals (Fig. 3A). The genetic diversity within both populations was similar (average *p*-distance values of 2.1 ± 2.6 in MC vs. 1.2 ± 1.9 in GB). Figure 3B maps the location where polar bears carrying the two most frequent haplotypes (*a* and *e*) were captured; again showing how individuals carrying the *e* haplotype are more common in GB and those with the *a* haplotype are mainly found in MC.

**Figure 4:**
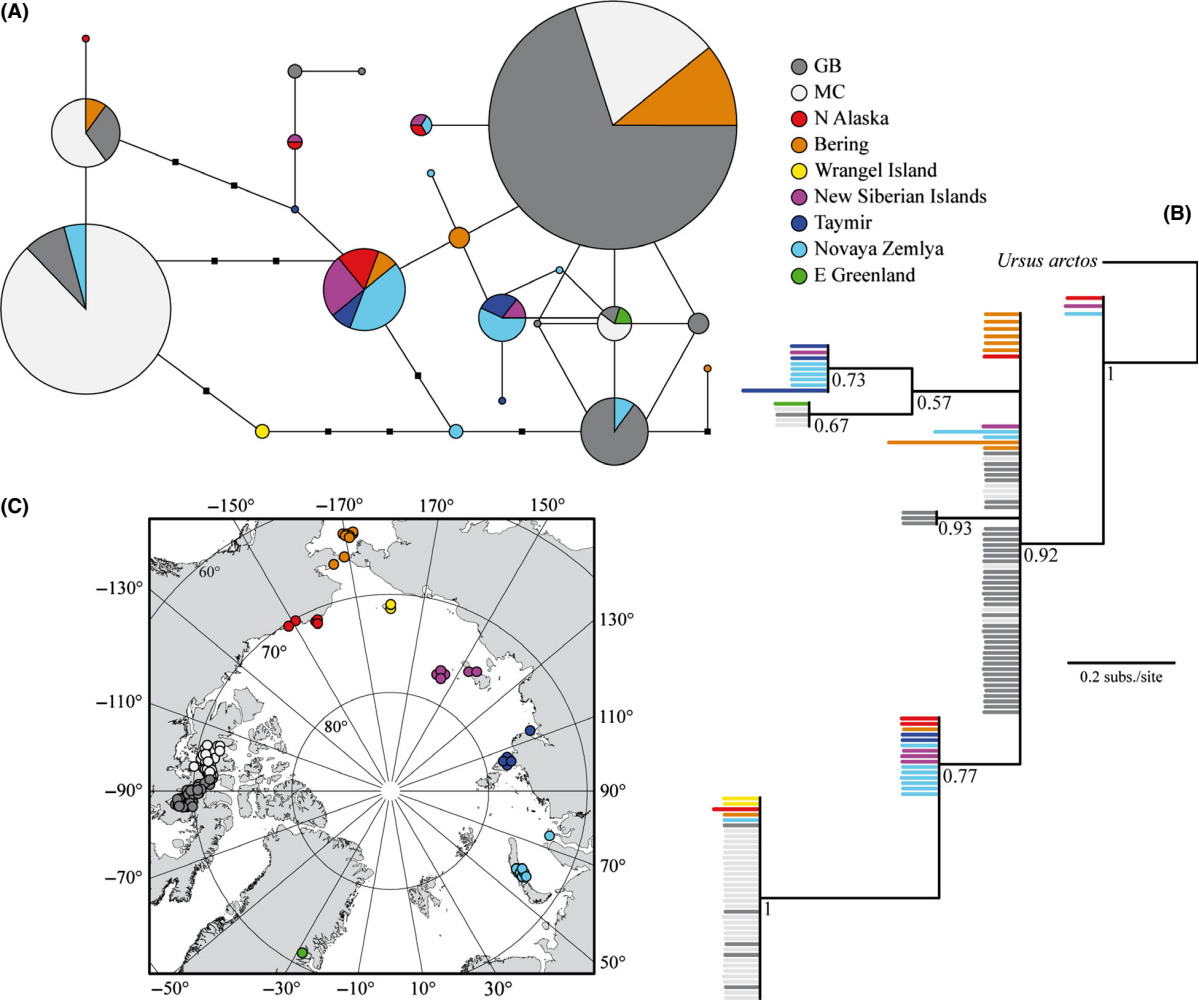
Phylogeographic analysis of polar bears using CR sequences. (A) Maximum parsimony network showing 95% probability linkages among 22 CR haplotypes obtained from 131 individuals sampled across the species’ range, including sequences from Edwards et al. (2011). Localities were grouped according to geographic proximity (for simplicity in displaying the results) and color coded in the network. Other details as in Figure 3A. (B) Bayesian phylogenetic topology with posterior probabilities indicating node support. Individuals are color coded by locality as in Figure 4A. (C) Map showing sampling localities.

The divergence between individuals from GB and MC is comparable to the divergence among CR sequences from individuals sampled across the species’ range (Fig. 4). When the CR data were reanalyzed together with 45 new sequences obtained from sites shown in Figure 4C, the statistical parsimony analysis (Fig. 4A) identified 22 CR haplotypes separated by up to 11 mutational steps. Thus the diversity in CR sequences observed at the scale of the central portion of the Canadian Arctic Archipelago is similar to the species CR genetic variation as a whole (compare the haplotype networks in Figs. 3A, 4A). Neither the network analysis nor the Bayesian tree (Fig. 4B) reveals a completely resolved genealogical structure. Haplotypes from the same or adjacent localities are often scattered throughout the parsimony network in Figure 4A. Although support for nodes in the Bayesian tree was low (generally <0.95) suggesting more data are needed to obtain a fully resolved topology, individuals from the same locality can be found in nearly every clade (Fig. 4B). The only highly supported clade within the species (posterior probability of 1.00) includes individuals from six of the nine localities sampled (Fig. 4B).

### Bayesian coalescent simulations

Estimates of the demographic parameters from the IM model differed between our mtDNA and nuDNA analyses (Table 1). Analyses of the mitochondrial data suggested that the effective population size is approximately fivefold larger in MC than in GB, while the DNA microsatellite data implied a threefold size difference in the opposite direction (Table 1). The latter trend was also obtained when effective population size was estimated using DNA microsatellite data and an Approximate Bayesian computation framework in *ONeSAMP* (average and 95% confidence interval limits: GB, 421 [371–555] individuals vs. MC, 80 [66–111] individuals). *IMa2* analysis of mtDNA suggests that there have been significant levels of gene flow since the establishment of the GB and MC populations (95% highest posterior density interval – 95% HPD – does not overlap with zero), whereas the converse is true when the analysis was performed using nuclear data (Table 1). Although estimates of splitting time from the IM model must be interpreted with caution due to the lack of species-specific molecular clock calibrations, we can use calibrations from other large mammal species. Using radiocarbon-dated samples and DNA sequences, Saarma et al. (2007) estimated the brown bear (*U. arctos*) CR to diverge at a rate of 29.8% (confidence interval: 13.3–47.6%) per million years. Huang et al. (2002) utilized known human pedigrees to infer a mutation rate of 1 × 10^−4^ per generation for dinucleotide microsatellites in *Homo sapiens*. Assuming that these mutation rates are comparable to those in *U. maritimus*, and using a generation time of 5 years (approximated through measurements of age of first reproduction of females in the study area, Taylor et al. 2006, 2009), we obtain an approximately 40-fold difference in the estimation of the splitting time model parameter between populations for the two classes of markers. Our analyses suggest differences in mtDNA haplotypes began accumulating in the Holocene (*ca*. 3500 years before present), while nuDNA implies GB and MC began to diverge very recently, *ca*. 85 years ago (95% HPD: 14–350 years ago). Posterior density curves for parameters estimated in the *IMa2* analyses and details for the calculations regarding the splitting time parameter are presented in Appendix S2, S3, respectively. An unlikely high divergence rate of *ca*. 1200% per million years or an extremely low mutation rate of 2.5 × 10^−6^ per generation (for the CR and microsatellites, respectively) would be required for time estimations to be congruent (Appendix S3). The posterior density curve for the *t*μ parameter estimated using mtDNA shows a sharp peak but does not return to zero at the upper bound of the prior (Appendix S2). We interpret this as representing a range of times for the establishment of the matrilineal structure that are equally probable and thus our estimate (obtained using the peak value) is conservatively recent. Estimates of effective population sizes and gene flow were similar when male and female microsatellite data were analyzed separately; however, values of *t*μ differed (Table 1). While the 95% HPD interval for *t*μ in males overlaps with zero, the estimate for females is similar to the value obtained when the data were pooled (Table 1).

**Table 1:** Demographic population parameters estimated using *IMa2*; population size parameters (θ = 4*Nμ*, where *N* is the effective population size) for GB, MC, and the ancestral population, splitting time multiplied by the mutation rate (*t*μ), and effective number of migrants per generation (2*Nm*)

	θ_GB_	θ_MC_	θ_Ancestral_	*t*μ	2*Nm*
mtDNA	0.38 [0–4.43]	2.08 [0.08–38.08]	10.73^1^	0.14^1^	1.59 [0.12–29.62]
nuDNA sexes combined	2.98 [0.43–12.18]	0.93 [0.13–4.18]	7.28 [4.93–11.18]	1.72 × 10^−3^ [2.8 × 10^−4^–7 × 10^−3^]	0.12 [0–19.7]
nuDNA females	1.88 [0.13–13.57]	0.33 [0.08–2.18]	6.48 [4.53–8.68]	2.8 × 10^−3^ [1.2 × 10^−4^–0.01]	1.91 [0–15.69]
nuDNA males	1.73^1^	0.33^1^	7.23 [5.08–9.43]	4 × 10^−5^ [0–0.03]	0.84 [0–20.40]

Values where the posterior probability peaks as well as 95% highest posterior density intervals ([95% HPD]: the shortest interval that contains 95% of the posterior probability) are shown. Parameters were calculated independently using mtDNA and nuDNA, and for the latter we obtained estimates for both sexes separately and combined.

1 Posterior density reaches lower values but not 0 near the upper or lower limit of the prior.

## Discussion

Our analyses of mitochondrial and nuclear sequence data suggest that polar bears from the GB and MC do indeed comprise genetically differentiated populations consistent with previous designations (Taylor and Lee 1995; Bethke et al. 1996; Paetkau et al. 1999; Taylor et al. 2001), albeit at low levels that imply ongoing gene flow and/or recent common ancestry. Although analyses of both mtDNA and nuDNA support this assertion, these distinct classes of molecular markers reveal signals of divergence at different temporal and spatial scales. We found significant differences in CR haplotype frequencies between the GB and MC populations, and mtDNA structure common to individuals from across the species’ range (Fig. 4) that most likely originated elsewhere before or during the Holocene. In contrast, the differentiation in DNA microsatellites dates to modern times, possibly reflecting contemporary movement patterns, changes in the species distribution, and impediments to gene flow that have resulted from recent changes to the Arctic landscape (e.g., changes in the ice conditions that influence hunting opportunities). Below we discuss our findings in the context of the questions that motivated this study.

### Population differentiation across multiple temporal and spatial scales

Paetkau et al. (1999) found the GB and MC populations to cluster with others from the Canadian Arctic Archipelago (Lancaster Sound and Viscount Melville Sound) and the two adjacent populations to the east (Baffin Bay and Kane Basin). Paetkau et al. (1999) reported an *F*_ST_ value of 0.011 between GB and MC, remarkably similar to the estimate of 0.01 that we found with approximately eight times more sampling effort but a subset of the microsatellites (nine out of 16). The differentiation between our two focal populations is moderate compared to that found by Paetkau et al. (1999) over a broader spatial scale: pairwise *F*_ST_ range 0.002–0.108. Census population sizes estimated using mark-recapture data were 1523 ± 285 individuals for GB and 284 ± 59 bears for MC (Furnell and Schweinsburg 1984; Taylor et al. (2006, 2009); Obbard et al. 2010). Frankham (1995) found effective population sizes (*N*_e_) to be often markedly smaller than census population sizes (*N*), particularly for bears where *N*_e_/*N* was between 0.28 and 0.69. In a study conducted on polar bears from the Beaufort Sea (Alaska), Cronin et al. (2009) found *N*_e_/*N* to be ∼0.182. Similarly, our estimations of effective population size using approximate Bayesian computation suggest a *N*_e_/*N* ratio of 0.28 for both GB and MC (*N*_e_/*N*: 421/1523 vs. 80/284). Thus, with relatively small effective population sizes (especially for MC where polar bear numbers were reduced drastically by overhunting) it is likely that genetic drift has played a role in shaping differences in allele frequencies. Although the results from our *IMa2* analysis must be interpreted carefully, the data suggest that the genetic differences between these populations arose within the last hundreds of years. This time frame suggests that the land barrier that separates GB and MC (the Boothia Peninsula) is sufficient to at least partially restrict polar bear movement across these areas, and that the Bellot Strait does not constitute a corridor connecting GB and MC. We agree with Paetkau et al. (1999) that it is surprising that an animal with such a striking capacity to travel long distances would show genetic evidence of restricted movements across relatively small geographic areas. As Paetkau et al. (1999) hypothesized, this is possibly a consequence of sea ice and prey distribution. Additionally, the fidelity females show to denning areas (Amstrup and Gardner 1994; Zeyl et al. 2010) could play a role in shaping population differentiation.

In their broad-scale study of the origin of modern polar bears, Edwards et al. (2011) found few highly supported nodes (posterior probability >0.85) within a CR clade comprising ∼50 ancient and modern polar bears. Despite sampling individuals throughout the species’ range (see Fig. 4C), their study did not find an unequivocal phylogeographic pattern. Both Edwards et al. (2011) and Hailer et al. (2012) have suggested that multiple hybridization events occurred between brown bears and polar bears throughout the Pleistocene, and that mitochondria have introgressed from the former to the latter species. Thus, it is possible that such mtDNA introgression confounded our attempts to infer polar bear demographic history. Using CR sequences from GB and MC individuals we found marked genetic differentiation between our inferred focal populations (Φ_ST_ = 0.47, *F*_ST_ = 0.29), yet no clear phylogeographic pattern when compared to the CR sequences obtained by Edwards et al. (2011). This raises the interesting question of how such frequency-based differences in mtDNA between GB and MC arose when population structure seems to be absent at a larger scale. Edwards et al. (2011) interpret the distribution of modern bear matrilines as the product of long periods of stability in local conditions interrupted by rapid dispersal events and occasional hybridization with brown bears. Thus, the differences we observed in our mitochondrial data could have originated as populations became isolated in different glacial refugia, as has been suggested to explain mitochondrial genetic patterns in other Arctic species (e.g., rock ptarmigans, Holder et al. 1999; collared lemmings, Federov and Stenseth 2002). A possible explanation for the mitochondrial differentiation apparent between GB and MC populations is that it is the product of a founder effect. These areas could have been colonized by small groups of individuals possessing mitochondrial haplotypes that diverged elsewhere; fine-scale differentiation was produced by random sorting of mtDNA diversity that existed at the time of colonization. Phylogeographic studies of other Arctic species suggest modern ranges are the product of postglacial expansions from multiple glacial refugia where populations had remained isolated (e.g., Holder et al. 1999; Federov and Stenseth 2002). Ultimately, more intensive geographic sampling across as many polar bear populations as possible, combined with phylogeographic data from an array of species with divergent life histories (especially less vagile species) will help us understand the dynamic history of polar bear populations.

Four lines of evidence suggest that the mitochondrial and nuclear genetic patterns observed between GB and MC differ in origin. First, our *IMa2* estimations of effective population sizes inferred through mtDNA and nuDNA are contradictory. While mtDNA suggests the effective population size of MC is approximately fivefold larger than that of GB, nuDNA shows a threefold difference in the opposite direction (there is a fivefold difference in favor of GB when *N*_e_ is estimated with *ONeSAMP* using nuDNA). Direct estimates of current census population sizes in the study area (Furnell and Schweinsburg 1984; Taylor et al. (2006, 2009); Obbard et al. 2010) indicate that in fact the population of GB is roughly fivefold larger than that of MC. Assuming effective population sizes are proportional to the absolute number of individuals in the population (although generally lower; Frankham 1995), then this is consistent with the estimates obtained using nuDNA that show larger effective population sizes in GB, supporting our interpretation that mtDNA reveals differentiation at a different time scale within the species. Second, while our *IMa2* analysis using mtDNA shows that the GB and MC populations have experienced gene flow since their establishment, the nuclear data imply that the contrary is true. A third line of evidence comes from the geographic scale at which nuDNA and mtDNA genetic divergence is observed. While DNA microsatellites reveal differentiation at a local scale (e.g., the study by Paetkau et al. (1999) shows higher *F*_ST_ values when comparing geographically more distant populations consistent with population-level isolation by distance sensu Wright 1943), the differentiation in mtDNA is common to individuals across the species’ range. Finally, our *IMa2* estimates of divergence times are also discordant. Although the limited differentiation of nuDNA suggests that these populations were established during the last century, the divergence in mtDNA probably dates to before or during the Holocene. Edwards et al. (2011) dated the matrilineal ancestor of all extant polar bears (not only those from MC and GB) to 51,000–20,000 years before present; this coalescence time implies that the genetic diversity observed in our CR sequences may be much older than we found, perhaps consistent with the long tail in the splitting time distribution. Again, our estimates of divergence times are approximate as *IMa2* assumes that all populations that are exchanging genes have been sampled and this is clearly not true of our surveys. Our analyses did not include all polar bear populations interacting in the model at the same time; however, conducting analyses across the species’ range with the sampling intensity we have achieved for MC and GB would be both logistically challenging and computationally intense. Strasburg and Rieseberg (2010) found *IMa* is robust to small or moderate violations of the IM model assumptions, including introgression from an unsampled population. Nevertheless, future effort should focus on validating our findings by expanding sampling to the neighboring populations found by Paetkau et al. (1999) to be most closely related to GB and MC, and thus likely to be exchanging genes with them. In summary, our findings illustrate how nuclear and mitochondrial DNA markers may capture different aspects of the demographic history of a species. Thus, results from analyses of either class of marker considered alone may only reveal part of a species demographic and evolutionary history. Moreover, the combination of these markers in the same analysis must be interpreted with caution as they could potentially produce intermediate spurious results.

### Gene flow between GB and MC populations

Our indirect estimates of gene flow using *IMa2* suggest gene exchange has been low since the GB and MC populations began to differentiate. However, the effective number of migrants per generation (2*Nm*) calculated using CR sequences was significantly different from zero. As we argued above, we do not think that this represents the signature of contemporary gene flow between GB and MC, but rather the vestige of genealogical events that occurred elsewhere. Our estimates of the effective number of migrants per generation using nuDNA did not differ significantly from zero suggesting that contemporary gene flow between these populations is insufficient to homogenize them. This implies that low microsatellite differentiation and the admixture shown by the Bayesian assignment analyses (Fig. 2) is a product of recent separation rather than ongoing gene flow. Interestingly, there were three cases in which adult females initially caught in MC were recaptured in GB, two of which had a pair of cubs. We do not know if these represent real dispersal events or simply transitory movements of individuals between populations that did not lead to gene flow. Taylor et al. (2001) reported limited movement among populations north of our study area. For example, they found that only two of 65 bears for which there were multiyear satellite telemetry data available moved between defined populations among years. Annual rates of movement across population boundaries based on mark-recapture data were estimated at between 0.4% and 8.9%. Certainly, if the movement between MC and GB that we observed led to gene flow, their genetic contribution to the overall population was not sufficient enough to be evident in our *IMa2* analysis.

Finally, we found no compelling evidence for sex-biased dispersal or gene flow. Our mark-recapture data suggest that males and females do not differ in the distances traveled between years. The *IMa2* results did not show evidence of gene flow between populations either for analyses with sexes combined or when analyzed separately. These results are consonant with those of Taylor et al. (2001) who did not find differences in distances moved between sexes in six more northerly Arctic polar bear populations. Zeyl et al. (2009) combined microsatellite and field data to investigate spatial patterns of relatedness and dispersal in polar bears of the Barents Sea. Their analysis of genetic relatedness of same-sex dyads revealed stronger kin structure in female dyads compared with male dyads. Zeyl et al. (2009) concluded that at broader spatial scales effective dispersal was slightly male biased, and thus that male-biased gene flow may preclude genetic differentiation. We found that females had stronger signal of nuDNA differentiation between MC and GB, both in our Bayesian clustering analyses and reflected in females showing larger estimates of *t*μ in the *IMa2* analyses. This result would be consistent with male-biased gene flow or could simply be spurious, reflecting the lower sample sizes for males.

### Conservation and management implications

Both nuDNA and mtDNA show that polar bears from the Canadian Arctic Archipelago spanning GB and MC comprise two genetically distinct populations. The level of differentiation between GB and MC is moderate when compared to polar bears from other populations (Paetkau et al. 1999). However, our data suggest that gene flow is currently insufficient to homogenize these populations. These genetic differences imply that GB and MC should be treated as separate management units (sensu Moritz 1994) supporting previous findings (Taylor and Lee 1995; Bethke et al. 1996; Paetkau et al. 1999; Taylor et al. 2001). Our mark-recapture data suggest that individuals from MC travel on average larger distances than those from GB, although statistical significance was only achieved when the three females that moved between populations were included. This could reflect differences in the availability of high quality habitat for seals (Barber and Iacozza 2004) or fewer mating opportunities, the later being a consequence of the larger area and lower polar bear density of MC compared to GB (Obbard et al. 2010). Current efforts to monitor and model the demographics of GB and MC separately are justified and crucial to the recovery of the MC population from intense hunting. Future effort should include the neighboring populations in a more comprehensive genetic and ecological analysis that would provide a better understanding of the connectivity of polar bear populations in the Canadian Arctic Archipelago.

## Data Accessibility

Our microsatellite data set and all *Structure*, *Geneland*, *Arlequin,* and *IMa2* input files are available on the Dryad Repository (doi:http://dx.doi.org/10.5061/dryad.h1v63). Mitochondrial sequences were deposited in GenBank (Accession Numbers KF192517 – KF192602).

## Supporting Information

**Appendix S1.** Plots of the likelihood of the data for different values of *K* in the *Structure* analyses together with a table indicating the proportion of membership of each predefined population to the inferred clusters (when *K* = 2).

**Appendix S2.** Posterior density curves for the different parameters estimated using mitochondrial (CR sequences) and nuclear (DNA microsatellites) data in *IMa2*.

**Appendix S3.** Calculations of the splitting time parameter in years using information from *IMa2* runs and approximate molecular clock calibrations for CR sequences and dinucleotide DNA microsatellites.
