# Supplementary figures and images for: Extensive sampling of polar bears (Ursus maritimus) in the Northwest Passage (Canadian Arctic Archipelago) reveals population differentiation across multiple spatial and temporal scales
Source: Ecol Evol. 2013 Aug 3;3(9):3152–65. doi: 10.1002/ece3.662 (PMC3790558; doi:10.1002/ece3.662)

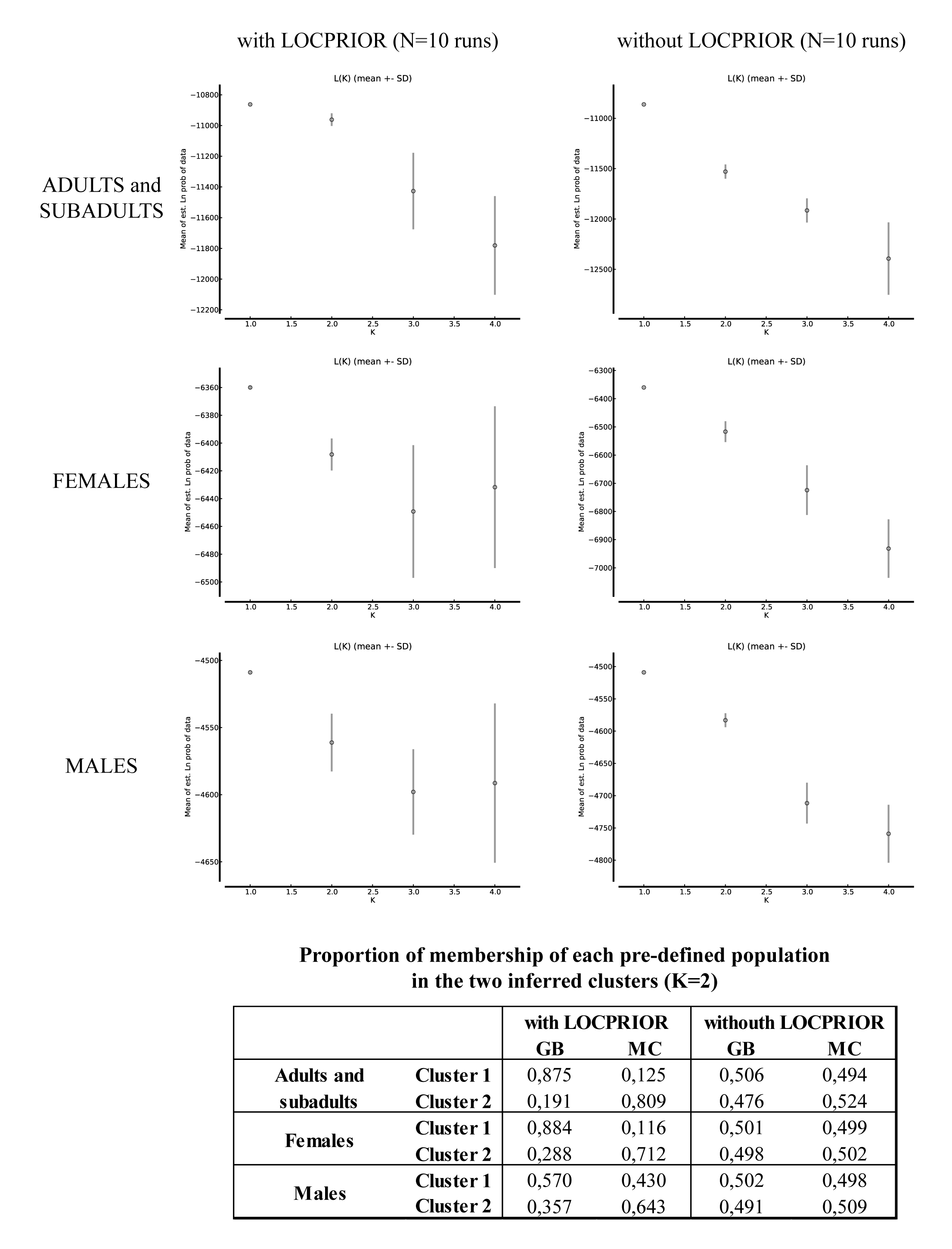

Supplement: Supplementary file 1 [file ece30003-3152-SD1.tif]

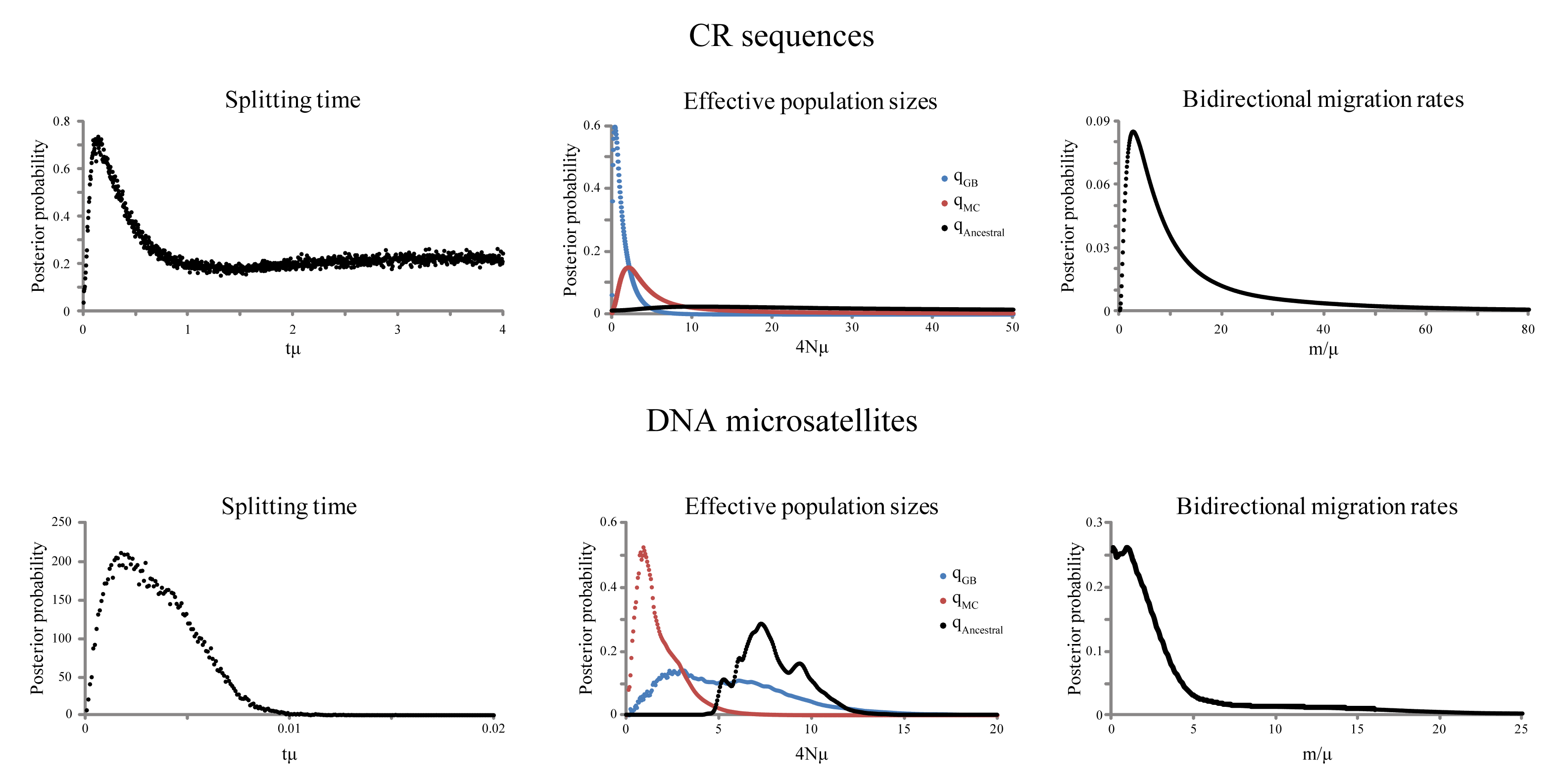

Supplement: Supplementary file 2 [file ece30003-3152-SD2.tif]
